# Monitoring Upper Extremity Function of Individuals With Breast Cancer: Development and Usability of the *StrongArms-Cancer* mHealth System

**DOI:** 10.1177/00469580261441759

**Published:** 2026-04-10

**Authors:** Jenna Smith-Turchyn, Jordon L. Hvizd, Edward R. Sykes, Som D. Mukherjee, Tara Packham, Margaret L. McNeely, Carolyn Moorlag, Ethan Shen, Haruna Isah, Christopher Anand, Julie Richardson

**Affiliations:** 1 McMaster University, Hamilton, ON, Canada; 2 University of Guelph, ON, Canada; 3 University of Alberta, Edmonton, AB, Canada; 4 Sheridan College, Oakville, ON, Canada

**Keywords:** breast cancer, upper extremity function, lymphedema, usability testing, mHealth

## Abstract

Upper extremity (UE) impairment is a leading cause of decreased function in breast cancer (BC) survivors. Failure to recognize and properly manage these impairments can lead to significant and sustained functional limitations. Few supports exist to help survivors monitor UE impairments related to BC over time. The purpose of this project was to evaluate usability of the *StrongArms-Cancer* mHealth system from the patient and the health-provider perspective. This project had 2 parts to evaluate the usability of the *StrongArms-Cancer* system. Part 1: Participant usability testing; Part 2: Expert heuristics evaluation. In part 1, participants were adults with a previous diagnosis of BC. They were asked to use the think-aloud method while completing 6 tasks in the system to assess usability. Users completed the mHealth App Usability Questionnaire (MAUQ) and System Usability Scale (SUS) at the end of the session. Session transcripts were coded using deductive content analysis to identify specific types of usability issues. Quantitative data from the MAUQ and SUS were summarized using descriptive statistics. Participants in part 2 were health providers living in Canada. They were asked to review and evaluate the mHealth system based on established heuristic principles. Data from the heuristic evaluations were summarized descriptively into main usability issues identified. Think-aloud transcripts highlighted 10 categories of usability issues. Most users (n = 12; 75%) rated the system as “excellent” or better; the mean SUS score was 86.9 (SD 12.06), demonstrating a “superior” score. MAUQ total scores averaged 104.7 (SD: 14.91). The 2 heuristic criteria that resulted in the greatest number of violations (n = 5) were “help and documentation” and “consistency and standards.” The findings from think-aloud sessions and heuristic evaluations revealed high user satisfaction and demonstrated effective usability. The *StrongArms-Cancer* mHealth system shows promise in aiding the proactive surveillance and monitoring of individuals with BC.

## Background

In 2024, approximately 30 500 Canadians were diagnosed with breast cancer (BC).^1^ Fortunately, the 5-year survival rate for BC is approaching 90%.^2^ However, survivors live with many physical and psychosocial treatment-related side effects for years after treatments have ended.^3,4^ Upper extremity (UE) impairment is a leading cause of decreased function in BC survivors.^5,6^ Lymphedema affects approximately 40% of survivors and impaired shoulder range of motion (ROM) has a prevalence of up to 65%.^7-10^ Risk factors for lymphedema development and UE ROM impairment include breast surgery, lymph node dissection, radiotherapy, obesity, seroma formation following surgery, arm trauma, and chemotherapy.^11,12^ The large majority of BC survivors possess 2 or more of these risk factors.

While risk of lymphedema and UE ROM limitations are high, these conditions are preventable, even reversible, if identified and treated early.^6^ The current model of healthcare in Canada relies on the identification of significant arm swelling or UE ROM impairments by a health professional to diagnose these impairments, but systematic screening is lacking. It delays diagnosis, compounds impairment, and complicates management.^13,14^ Failure to recognize and properly manage these impairments can lead to significant and sustained functional limitations.^6^ This further leads to both decreased quality of life and decreased participation in meaningful activities at home and at work.^4-6,8^ Self-care strategies are needed to facilitate a pro-active and productive approach between rehabilitation and chronic condition management.^15-17^ Self-care and self-management strategies empower individuals to actively manage their own health issues; preventing the development of secondary and tertiary disability, enabling better self-monitoring capability that is more accessible and will allow for monitoring of more incremental changes, and promoting motivation for and adherence to intervention regimes.^18-20^ Self-care strategies may also reduce healthcare costs.^18-20^

Barriers to systematic and timely monitoring of UE impairment in survivors of cancer by health professionals include a lack of (1) patient access to health professionals with knowledge of UE function, (2) clinical time and resources, and (3) patient time due to competing life demands.^21^ Additionally, the volume of survivors makes it difficult for the current system to regularly assess and manage UE function. This is especially true as the incidence of these impairments increases with time post active treatment (eg, lymphedema incidence increases from 13.5% at 2 years to 41.1% at 10 years post treatment).^7^ During this period survivors see their oncology team less frequently, compared to during treatment, if at all. Providing accessible self-care strategies is vital as the incidence and burden of cancer is projected to increase substantially over the next decade.^22^

Recent studies suggest that self-measurement of arm circumference using a tape measure by women with and without lymphedema is reliable.^23,24^ However, the method used had survivors manually measure and record arm circumference and has several limitations including the need for patient training by qualified health professionals on how to measure the arm properly. Further, this method poses concerns due to the potential for random error during the measurement process *and* when recording scores *over time*.^23,24^ This process also required users to interpret measurements over time and decide on appropriate next steps if changes occur. This can lead to further error as the mean clinically important difference (MCID) for circumferential measurements is small, at 2cm.^24^ Incorrect interpretation can lead to missed or delayed detection of impairment onset, and more severe long-term limitations. Further, patients cannot perform UE ROM measurement using goniometry unassisted.

To decrease measurement error and improve reliability as well as consistency, technology use has been encouraged,^25,26^ however, few supports exist to help survivors monitor UE impairments related to BC over time. Technology increases accessibility and availability of rehab services and promotes self-management of chronic conditions,^26,27^ and survivors of cancer demonstrate need, acceptability and adherence to rehabilitation programs using technology.^28^ Current self-care apps available for survivors of cancer provide survivorship education (eg, resources related to physical activity), social networking (eg, a platform to interact with peers), and symptom tracking (eg, self-recorded health observations).^29^ However, no mHealth system currently manages UE function over the short and long term. Furthermore, a criticism of currently available apps is that they leave the user with the task of weaving together pieces of information to interpret and track over time.^29^

While tape measure apps^30,31^ exist, their reliability and validity are not well explored and current ones do not measure UE circumference or volume, which is needed to identify lymphedema. Goniometry apps^32,33^ to measure UE ROM exist and demonstrate high inter and intra-rater reliability (ICC = 0.995-1.0)^34,35^ and concurrent validity (ICC = 0.998-0.999).^35-37^ However, existing apps (1) are not BC specific, (2) do not exist on a platform together, and (3) do not tailor evidence-based recommendations in response to the measurements obtained over time. Therefore, current systems do not provide accurate and usable information to identify and address UE impairment in this population. Accessible, accurate, and easy to use self-care tools using technology are needed for survivors to prevent complications, track UE function, and identify and deal with changes early if they do occur to prevent functional limitation.

### Objective

This paper describes the development of the *StrongArms-Cancer mHealth system* and reports usability testing results of this system. We had 2 objectives: (1) Evaluate usability, acceptability, satisfaction of the system from the patient perspectives using think-aloud methods and usability questionnaires, and (2) Evaluate usability from the health-provider perspective using a heuristic evaluation. Both patient and provider perspectives were explored to investigate differences based on experience and expertise, digital and health literacy, contexts of use and workflow considerations, and overall to improve future implementation success in real-world settings.

## Methods

### Study Design

This project had 2 parts to evaluate the usability of the *StrongArms-Cancer* mHealth system. Part 1: Participant usability testing; Part 2: Expert heuristics evaluation. The Hamilton Integrated Ethics Board approved this study (Study ID: 17090). The STROBE guidelines were used in reporting these findings.^38^

#### StrongArms-Cancer mHealth System Design and Development

##### Design Framework

The Information Systems Research (ISR) framework^39^ was used in the design and development of the *StrongArms-Cancer* mHealth system. The ISR framework is an iterative, non-linear process and has been used extensively in design science research.^39-41^ It has 3 research cycles: The Relevance Cycle (seek to understand users’ needs and environment), the Design Cycle (where artifacts are produced and evaluated) and the Rigor Cycle (artifacts become part of knowledge base, are evaluated, and different representations are formed to suit user needs).^39-41^

##### StrongArms-Cancer System Design and Development

The *StrongArms-Cancer* system was designed and developed using a combination of Experience-Based Co-Design and Design Thinking processes between October 2023 and September 2024. Experience Based Co-Design is an approach that enables patients and other stakeholders to co-design services together with researchers and developers.^42^ Design Thinking is an iterative process where developers seek to understand the user, challenge assumptions and define/redefine problems in an attempt to improve the design and usability of a service.^43,44^ It begins with a pictorial description of a product and as more detail is added, progresses to more complex representations.^41,43,44^

To begin development of the *StrongArms-Cancer* system, we met with target users (4 BC survivors) to discuss goals when using the system and potential barriers to use. They identified 4 goals of the *StrongArms-Cancer* system: (1) to provide accurate measurements, (2) include an easy-to-read graph of measurements over time, (3) be intuitively easy to use, and (4) provide easily understandable action items for prevention. Based on this information, we created wireframes and mock-ups to visualize the user experience and information architecture of the system.

Consistent with Design Thinking, we first created low-fidelity paper prototypes. In the next phase, with medium-fidelity prototypes, we used simple web apps to implement the flow of the *StrongArms-Cancer* system, without including motion sensors. Finally, we built high-fidelity prototypes using: (1) a cross-platform Progressive Web App (PWA; “App”) using motion sensors (ie, built-in device accelerometer and gyroscope sensors) and other advanced features, such as augmented reality, and (2) a measurement wand with a passive transducer and Bluetooth chip that relays measurements to the PWA on the user’s device. Table 1 provides an overview of development using ISR and Design Thinking.

**Table 1.** Development Process Using the ISR Framework and Design Thinking Methods.

ISR cycle	Design thinking mode(s)	Study process^a^
Relevance	Define empathize	● Initial understanding of user and problem: investigator meeting to discuss target users, system goals, potential barriers to achieving goals ● Visualize user experience: assess wireframes & mock-ups
Design	Ideate prototype	● Generate ideas and create prototypes (low, then medium, then high-fidelity based on user feedback at each stage) ○ Get feedback at each stage, update prototypes, and select from prototype options in response
Rigor	Test	● Evaluate the system: usability testing for application ● Refine / improve product, consider maintenance

a Please note this is an iterative process.

##### Development Procedure

**Figure 1.**
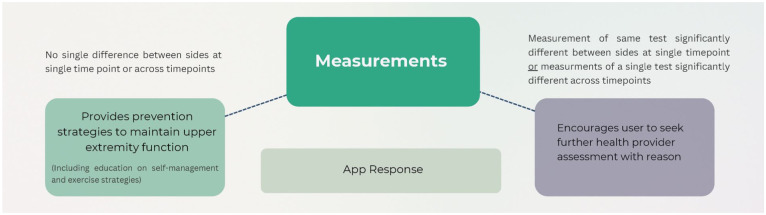
Overview of system response based on measurements obtained.

A team of multi-disciplinary experts (ie, physio therapists, engineers, computer science experts) from McMaster University and Sheridan College in Ontario, Canada created the *StrongArms-Cancer* mHealth system. It includes 3 components: (1) a cross-platform mobile application (App) that runs on all smartphones and tablets, (2) a Bluetooth-enabled measurement wand, and (3) a middle-tier component (server) which provides a portal to the system to store all confidential information in a secure and privacy-enhanced database. The system provides 2 core tools: (1) a digital goniometer (ie, an instrument for the measurement of angles; to measure UE ROM) and (2) a digital volume device (“measurement wand” to measure UE volume). The system measures and transmits the user measurements and tracks and records changes in measurement over time. Based on the measurements obtained and risk of impairment, the App triages evidence-based management strategies.^5,45^ If no impairments are noted and/or no significant change has occurred (ie, <5% difference on volume measurements or <7° difference on ROM measurements^46^), the App provides prevention strategies on the user’s device. If impairment and/or significant changes are noted (ie, ≥5% difference in volume measurements^24^ or ≥7° difference on ROM measurements^46^), the App summarizes findings and alerts the user to seek further medical attention. See Figure 1 for App response based on measurements obtained.

##### Application Design

**Figure 2.**
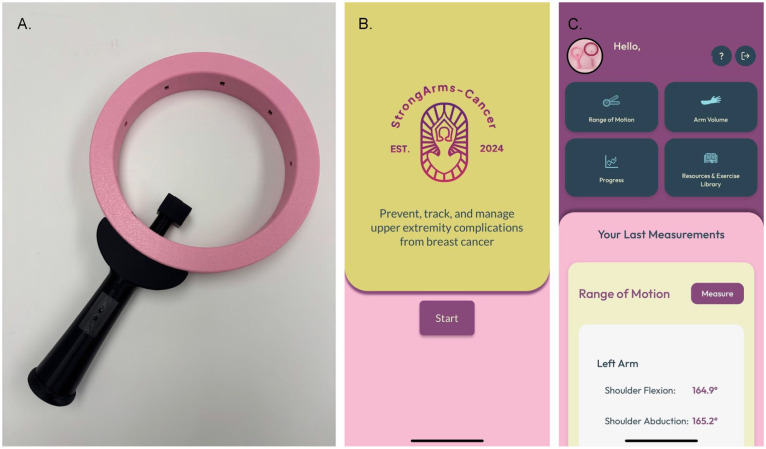
*StrongArms-Cancer* mHealth system: (A) measurement wand, (B) app log-in screen, (C) app home screen.

Within the App we focused on making the different functions easy to find and use, maximizing white space with minimalistic design to limit confusion, and providing obvious focus points on the page. Figure 2 provides a screenshot of the *StrongArms-Cancer* App interface and measurement wand. The development of the content in the App was built on evidence-based guidelines^5,45^ and reviewed by rehabilitation experts. Collaborators and end users were consulted throughout the development and design process to give their perspective.

#### Part 1-Usability Testing

##### Participants and Recruitment

To be included in the study, participants had to be: (1) English-speaking; (2) survivors of BC (any time post diagnosis); (3) ≥ 18 years; and (4) currently receiving or having completed treatment for primary BC. Participants were excluded if they self-reported any physical injury/condition that prevents them from completing assessments or had a diagnosis of a cognitive condition (ie, dementia) that precluded them from understanding instructions and/or the consent form.

Recruitment occurred between January and April 2025, primarily using advertising in local newspapers (ie, local “Coffee News” publications in Hamilton and surrounding area) and on social media. Additionally, a medical oncologist (SDM) at the Juravinski Cancer Center in Hamilton referred potential participants. Interested survivors emailed the Research Coordinator (RC). The RC connected with potential participants to determine eligibility, review the study information and consent form, and schedule an in-person usability session. At the session, participants signed the consent form and answered demographic questions regarding their age, sex, gender, presence of UE pain, and lymphedema status. All usability testing sessions were conducted in the Institute of Applied Health Sciences at McMaster University in Hamilton, Ontario.

##### Sample Size

The desired sample size for patient usability testing was 10 to 20 participants (or until saturation of data occurred). This was determined based on sample size suggestions for usability study designs, which suggests between 3 and 20 participants.^47,48^

##### Procedures & Settings

Participants were given instructions on system features and how to use the system at an individual in-person meeting with study staff at McMaster University. During this meeting participants were asked to use the think-aloud method^49^ while completing 6 tasks in the App to assess usability. The think-aloud method was developed in the field of cognitive psychology and has individuals verbalize their thoughts concurrently as they are performing tasks with the goal of assessing cognition at the same time as behavioral occurrences.^49^ This method is one of the most used tests of usability in healthcare and is associated with a further iteration of an App being developed.^49,50^ Usability participants were asked to perform 6 tasks: (1) log in (ie, enter username/password provided, read through introductory pages of App), (2) measure ROM (ie, watch instructional videos and measure using goniometer function for each UE measurement identified, including shoulder flexion, abduction, internal and external rotation), (3) measure arm volume (ie, watch instructional video and using *StrongArms-Cancer* wand measure arm volume from wrist to axilla), (4) access stored graphical data (ie, navigate to the progress graph to see first measurement, adjusting settings to look at 1 arm individually or both arms at the same time), (5) access resources/exercise library (ie, navigate to the resource section, look through the different resources, access the exercise library, and review the exercises provided), and (6) log out (ie, find log out icon and close down App). Participants were prompted to continue talking when performing each task. Each session took between 60 and 90 min. Users completed paper mHealth App Usability Questionnaire (MAUQ)^51^ and the System Usability Scale (SUS)^52^ at the end of the session. Results from this stage will inform system updates, which will be validated by participant review and approval, prior to beginning further testing. Based on previous literature, we a priori determined that SUS scores had to be 70 or greater,^53^ and MAUQ subscale and total score averages had to be ≥5/7^51^ to demonstrate adequate usability and proceed to further phases of testing.

##### Outcomes

mHealth App Usability Questionnaire (MAUQ): The MAUQ for standalone mHealth apps used by patients^51^ was used to assess usability of the *StrongArms-Cancer* system. This is an 18-item self-report measure, including three usability subscales (ease of use: 5 items; interface/satisfaction: 7 items; usefulness: 6 items).^51^ Each item is rated on a seven-point Likert scale (1 (disagree) to 7 (agree)). Items are totaled and an average is calculated; higher overall averages represent higher usability.^51^ This scale has been validated and has demonstrated high levels of reliability.^51^

System Usability Scale (SUS): The SUS was used to measure users subjective perception of a systems overall usaiblity.^52,54^ This tool evaluates includes 10 items (1 = strongly disagree to 5 = strongly agree). Reliability for this scale is 0.83 to 0.97 and the scale has demonstrated convergent validity,^55^ and has been validated as a usability metric.^56^ A seven-point adjective-anchored Likert scale was added, as suggested by Bangor and Miller,^54^ to interpret individual SUS scores and relate the scale score to an absolute judgment of usability.

##### Data Collection & Management

Participants were assigned a random identifier and created a unique study-specific log in email and password by the research coordinator. They signed into the *StrongArms-Cancer* system (via the App) using that identifier; no personal information was collected in the App during usability testing. Measured, de-identified data within the App was collected using the built-in mechanisms. Usability data using the think-aloud method was digitally recorded and transcribed verbatim. Transcripts were uploaded into NVivo15 software for analysis. The MAUQ and SUS scores were uploaded into the study Excel spreadsheet at the end of the session.

##### Analysis

Usability session transcripts were coded using deductive content analysis^57^ to identify specific types of usability issues. Transcripts were grouped into themes for each task based on 16 specific types of usability issues identified by Kushniruk and colleagues.^58^ Quantitative data from the MAUQ and SUS were summarized using descriptive statistics (means ± standard deviations for continuous data; frequencies/percentages for categorical data).

#### Part 2-Heuristics Evaluation

##### Participants and Recruitment

Participants for the heuristic evaluation included adults who were: (1) living in Canada; (2) English speaking; and (3) health providers (ie, Physiotherapists, Occupational therapists, Kinesiologists, Physicians) with experience working with individuals with a physical health condition. Potential participants were excluded if they were not a registered health provider in Canada or had not worked directly with an individual with a physical health condition. Participants for this phase of the study were recruited using convenience and snowball sampling, by sending recruitment emails out to colleagues with clinical experience working with individuals with acute and chronic conditions.

##### Procedures & Settings

Health provider participants were asked to review and evaluate the mHealth system based on established heuristic principles independently at their desired location.^59^ There were a total of 12 heuristics evaluated for 4 tasks within the App. Each evaluation was completed on a separate chart (see Supplemental File 2). The 4 tasks evaluated were: Task 1 = Measure ROM, Task 2 = Measure Arm Volume, Task 3 = Access Result Graph; and Task 4 = Access Resources. Within this chart evaluators highlighted any issues they faced when performing each task and the severity of usability issue (ie, the difficulty they had with each task, rated as: 1 = mild, 2 = moderate, 3 = severe) and commented on the issue. This took approximately 2 h to complete. Once completed, evaluators uploaded the completed documents into a secure OneDrive folder, hosted by McMaster University.

After reviewing the system, health provider participants also filled out a short survey in Microsoft word highlighting strengths and areas of improvement for the system, and which patients this would be beneficial for in clinical practice. Questions included: *What are the strengths of the app? What are the areas that could be improved? Which patients do you think might benefit from using the Strong-Arms Cancer mHealth system? Which patients would you recommend this mHealth system to?*

##### Analysis

Participant data from the heuristic charts was summarized descriptively (frequency of unique violations, reported as number (percentage) into main usability issues identified on the heuristic’s evaluation form. Qualitative responses to survey data were analyzed using content analysis^60^ into main themes that emerged from the data for each question.

## Results

### Part 1: Usability Testing

Demographic characteristics of study participants are presented in Table 2. All participants (n = 16, 100%) were female and identified as a woman. The mean age of participants was 63.25 years (SD 9.1 years; range 49-75 years). Four participants (25%) reported having ongoing arm pain while 5 (31%) reported intermittent arm pain. Three participants (19%) reported current lymphedema.

**Table 2.** Participant Characteristics (N = 16).

Characteristic	Values
Age (years), mean (SD)	63.25 (9.1)
Sex, n (%)	
Male	0
Female	16 (100)
Gender, n (%)	
Woman	16 (100)
Man	0
Arm pain present, n (%)	
Yes	4 (25)
No	7 (44)
Sometimes	5 (31)
Current lymphedema, n (%)	
Yes	3 (19)
No	13 (81)

Think-aloud transcripts highlighted 10 categories of usability issues: color, understanding instructions, workflow issues, graphics, layout, font, navigation, meanings of icons and terminology, applicability, and timeliness. The task “Access Resources” received the greatest number of usability issues. Across all tasks, the most common usability issues were workflow related with difficulties using the start/stop button when measuring ROM and finding the wheel speed sensor overly sensitive. Direct feedback included suggestions to using bolder breast-cancer related colors (eg, deep pink) to help users identify buttons more easily and include an audible indicator alongside the start/stop ROM function. Usability issue examples by task can be found in Table 3.

**Table 3.** Think Aloud Results Highlighting Usability Issues.

Task	Usability issue	Description	Example
Task 1: Login	Color	Users found the color difficult to identify icons during login	*“I think it needs to be more bolder.. the background needs to be lighter and.. what you need to do needs to be darker and bolder, like.. the actual things that you had to click on.. you want to be kind of like intuitive..”* (User 11, 60 years old)
Task 2: Measure ROM	Understanding instructions	User had difficulty knowing next steps	*“The home button was a little bit confusing, yes, and then, because I’m not sure when I go to finish. . .to close it, like, can I be moving my arm down so I see better or [do I] have to stay in that position.”* (User 9, 70 years old)
	Workflow issues	User had difficulty starting and stopping the measurements	*“I just couldn’t see where to push or anything when it was up there. When you put your arm at the top, it was hard to press the button. . .you can’t see that [whether or not] you have actually closed it.”* (User 9, 70 years old)
	Graphics	User had difficulty with the buttons	*“Some of the things could be bigger and bolder, like the “x” and.. where you press to start, and things like that.. like the buttons are the on the homepage, yeah.. larger font, for sure.”* (User 11, 60 years old)
	Layout	User found the measurements section visually cluttered	*“If I was to use this on a regular basis, I probably want to be able to delete the videos, [or close] so all I see is where I need to go to do the measurement, rather than have to always, constantly go through and look at [the videos].. simplify it a bit.”* (User 16, 62 years old)
	Color	User found it difficult to identify the buttons due to color	*“I think that the colors are kind of dark.. When I go up, I can’t distinguish between what that says and that says. Yeah [but if it was] fluorescent..”* (User 16, 62 years old)
	Color	User found it difficult to distinguish between the different ROM directions	*“Maybe you do that two different colors, because.. it’s very easy to scroll past it.. So each, each of the four different directions.. maybe.. different clear colors”* (User 16, 62 years old)
	Font	User found the font too small	*“I think this “x” up here.. the one in the top right corner should be bigger, more noticeable.. some of the things could be bigger and bolder, like the “x” and the and.. where you press to start.. larger font, for sure. The font size.. should be larger. . .”* (User 11, 60 years old) *“I’m older and I need reading glasses.. settings that you can use to.. make the text bigger.”* (User 7, 65 years old)
Task 3: Measure arm volume	Navigation	User had difficulty locating the arm volume measurement function	*“So, I have to go back up [to] “Arm”.. so [first] we have to go out of this one, right?”* (User 8, 72 years old)
	Meaning of icons and terminology	User had difficulty knowing what the icons represent	*“Is that it there? That’s a goniometer, right? Is it this one?”* (User 10, 47 years old) *“But the picture is the same as the four choices at the beginning.. This one does not [have] the text with it, but same symbol.. though I can’t remember. Like, that’s home.. that’s range of motion.. I don’t know what* that is*”* (User 15, 66 years old)
	Understanding Instructions	User had difficulty following the instructed steps to use the wand	*“Okay, because I was gonna say, I don’t remember hearing click it to begin.”* (User 13, 61 years old) *“Just press, press, measure, right? Oh, yeah, first did I press something?”* (User 7, 65 years old)
	Workflow issues	User found discomfort with the wand against their skin	“*It’s a part here* that is *scratchy on the skin.. You can feel it with your finger.. it’s not very comfortable.. around the wheel, where it’s making contact.. when you move your arm, you feel this one here.. It’s easy to use it, but.. just not so comfortable with the the actual device*.” (User 14, 68 years old) *“..it feels a little rough.. I don’t like that.. It is a bit rough there on the skin, maybe changing the wheel, or is it the surf[ace], the square part?*” (User 7, 65 years old)
	Workflow issues	User found the wheel sensor too sensitive	*“It’s like, you twitch, and.. oh [it activates the light], it’s too much.”* (User 10, 47 years old)
	Workflow issues	User found it difficult to stop the arm volume recording	“*It’s kind of awkward to. . .press that button when you’re done. . . if it’s right in your armpit. That’s why I was trying to adjust my fingers. . .so that I could get ready to press it. It might be better if it was on the other side.. because then you would see it.*” (User 11, 60 years old)
	Workflow issues	User had difficulty using the wand on their own	“*It’s a little awkward. Maybe, if someone else did it for me.*” User 12 “*I just felt my [arm] getting tired. . .the left arm was a little bit heavy with [the wand], holding it.*” (User 14, 68 years old)
	Graphics	User found the “connect to wand” button size too small	“*Probably a little bit larger, a little.. yeah.*” (User 3, 71 years old)
	Graphics	User found the videos visually cluttering	“*I would probably want to be able to delete the videos. . .so all I see is where I need to go to do the measurement. . .simplify it a bit. . .a scroll down menu. . .the* first *time it’s open and then. . .after that, like, it’ll just be closed by default. . .because then it’s cleaner [and] there’s not a lot of clutter.*” (User 16, 62 years old)
	Graphics	User found it difficult to see where to close the video pop-up window	“*Changing that “x” to “close,” because I couldn’t see an “x” [there]. . .It was so small. It just looked like a little round thing. It doesn’t look like an X.*” (User 3, 71 years old)
Task 4: Check progress graph	Meaning of icons and terminology	User had difficulty knowing which icon to select	“*Once you get used to the icons, it’d be pretty good. But until you get used to it, it’s a little. . .it was a little confusing. Maybe you have some words.*” (User 4, 49 years old)
	Layout	User had difficulty with the scroll down menu to find the results for each arm	“*I didn’t realize I had to click on the left arm to get the right arm. . .[if] there’s a word there or something to say ‘select which arm. . .*” (User 5, 70 years old)
	Applicability	User wanted to see normal ROM values in the graph to interpret measurements	“*What is that? How do I do the interpretation? There’s no real interpretation right now. Don’t have a min or max.*” (User 2, 72 years old)
Task 5: Access resources and educational information	Graphics	User wanted to see visuals (images or videos) to accompany the resource articles	“*I think this last section, where it talks about posture, [you could] maybe put in some pictures or videos.. for people who are more visual.*” (User 10, 47 years old)
	Layout	User found the location and size of the navigation buttons in the resource section visually obstructive	“*So, because of the location of the buttons at the bottom of the screen, we can’t get to the last exercise. That’s a little bit bulky, the size the icons, just where the location is.*” (User 1, 75 years old)
	Applicability	User wanted more guidance in exercise resources	“*[I’d like it to] have a little bit more info about. . .what’s the recommended weight to start at, or how someone determines that for themselves. And then, like, how a person should go up in increments. . .how do you increase the weights? How much can I lift? This will differ for everyone, but maybe even, like, some kind of suggestion on what to start with, and if this feels too heavy, go to this.*” (User 10, 47 years old)
	Applicability	User would like to see more information on commonly prescribed medications	*“I’ve been taking medication for five years now. Every day [since] my surgery, and they tell me I have to do it for 10* *years. . .medication sometimes results in more injury. . .bone diminishes and all that stuff. So yah, maybe [medication] might be something to include”* (User 12, 55 years old)
	Applicability	User wanted to see instructional videos in the exercise resources	“*Have somebody talking you through it. You know, the things that you might forget, you know, even just like bringing your arm up, make sure that your arm is level with your shoulder. Yeah, and videos, not just like acting it out, but also pointing out, like small mistakes and why you you’d move in this direction, because it’s what it’s helping. You don’t even have to be that technical, but it’s going to help the muscles that need strengthening in your shoulder.*” (User 3, 71 years)
	Applicability	User wanted to see information on frozen shoulder	*“Has anybody brought up frozen shoulder to you? My sister, who [also] had breast cancer, had frozen shoulder. I’ve never had it. . .[but] it’s common with surgery. . .having some information on it in there [would be good].”* (User 3, 71 years old)
	Applicability	User wanted to see a chat or interactive Q&A function	“*A question answer and just say, ‘hey, has anybody felt this?’ A thread, and then you just go, okay, newly diagnosed, okay, I’ll go over here. . .it just gives a lot of positivity. Someone can search. . .maybe someone’s already asked this, right? You can post if you want, if you have a question, propose it there, and somebody can answer it. Or you go into a room and maybe you have the answer, yeah, because I just find that the breast cancer community, it’s [about] shared experiences, community. . .[it’s an] incentive to use the app as well. . .[makes it] more interactive.*” (User 7, 65 years old)
	Timeliness	User found resources were lengthy	“*It was not a lot to read, but a lot to understand. Yeah, so that’s the thing. It’s like. . .I don’t mind reading, and to have it where you completely understand every word for word that makes. . .the process easier.*” (User 4, 49 years old)
Task 6: Log out	Navigation	User had difficulty finding where to click to logout	“*That was hard to find. . .it did take me some time, because I went to this question mark here [instead].”* (User 7, 65 years old)
	Meaning of icons and terminology	User had difficulty knowing what the icons meant to logout	“*. . .The button didn’t say log out or anything, right? So, yeah, it would have been helpful if [it did].*” (User 5, 70 years old)

RA = research assistant; ROM = range of motion.

Most users (n = 12; 75%) rated the system as “excellent” or better; the mean SUS score was 86.9 (SD 12.06). This demonstrates a “superior” score as systems with a score greater than 70 are considered acceptable, scores in the high 70s to high 80s are considered superior.^54^ Four users (25%) reported the system overall as “best imaginable.” MAUQ total scores averaged 104.7 (SD: 14.91) with overall average MAUQ item scores at 6.5/7. Table 4 provides details of SUS and MAUQ score results.

**Table 4.** SUS and MAUQ Score.

Participant ID	SUS total score (/100)	SUS rating	MAUQ total score (/126)	MAUQ question average score (/7)
1	97.5	Excellent	110	6.89
2	92.5	Best Imaginable	119	7
3	95	Excellent	102	6.8
4	75	Excellent	116	6.82
5	67.5	OK	97	6.47
6	100	Excellent	119	7
7	95	Best Imaginable	112	7
8	80	Best Imaginable	117	6.5
9	62.5	Good	81	5.4
10	75	Good	101	5.61
11	100	Excellent	120	6.67
12	92.5	Best Imaginable	96	6.86
13	90	Excellent	94	5.89
14	100	Excellent	126	7
15	80	Excellent	77	5.5
16	87.5	Good	88	6.77
Average	86.9		104.7	6.5
St Dev	12.06		14.91	0.57

### Part 2: Heuristics Evaluation

**Figure 3.**
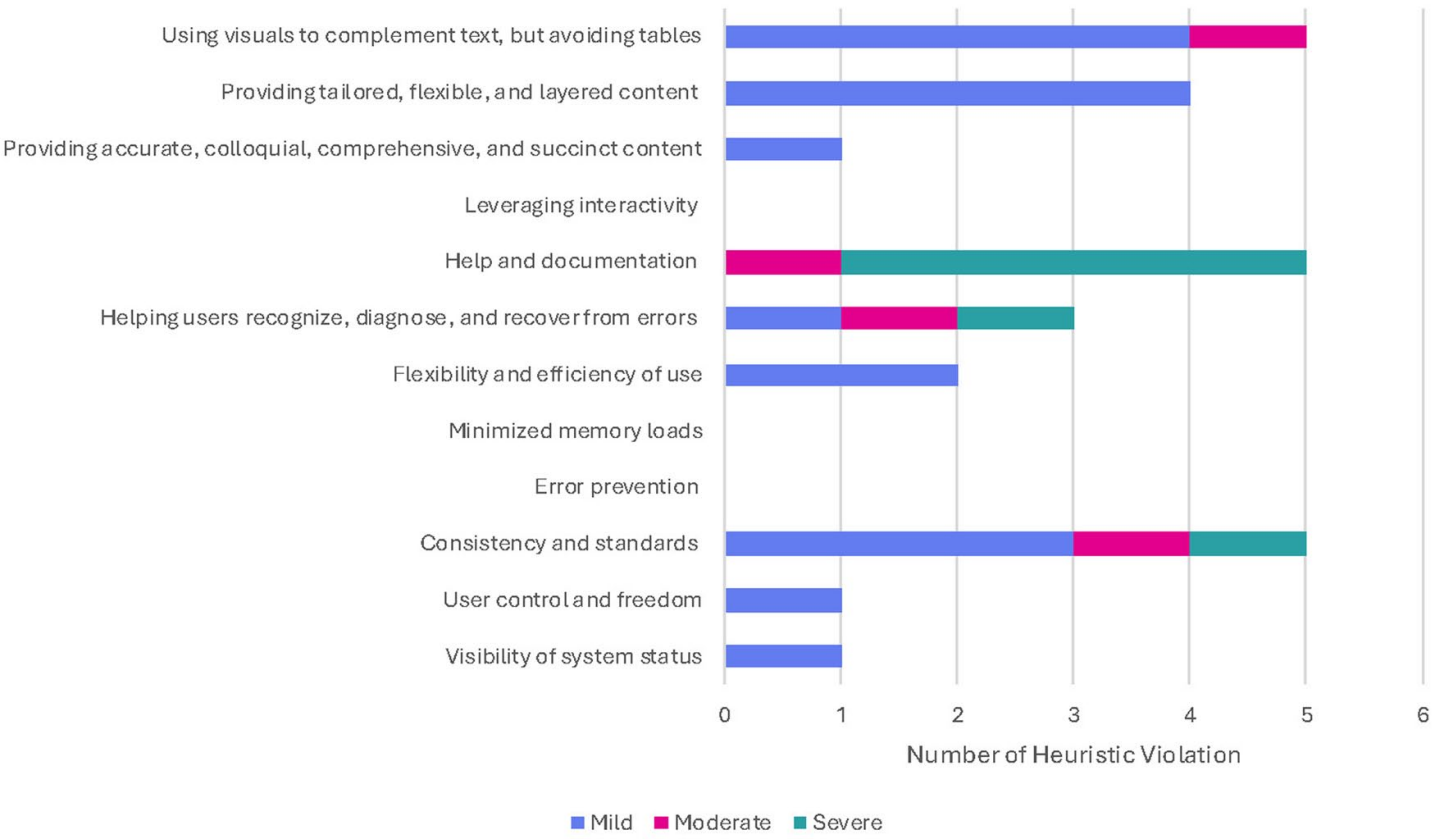
Frequency of heuristic violations by severity.

The heuristic evaluations were performed by 6 clinicians (4 physiotherapists and 2 kinesiologists). Figure 3 provides the overall frequency of heuristic violations for all tasks.

Overall, the 2 heuristic criteria that resulted in the greatest number of violations (n = 5) were “help and documentation” and “consistency and standards.” Results highlighted a need for a frequently asked questions (FAQ) information section, and revealed that the “?” icon at the top of the App did not function. This was followed by the heuristic criteria “help users recognize, diagnose and recover from errors” (n = 3). Raters suggested the system may detect and produce error messages for measurements outside of the normal range and would not detect when a measurement is altered by compensation from other joints. Mild violations were noted in each task under the heuristic criteria of “providing tailored, flexible, and layered content.” To overcome this, the recommendation was to have information available in more than one language. No violations (n = 0) were reported for the heuristic criteria of “leveraging interactivity,” “minimized memory loads,” and “error prevention.”

**Figure 4.**
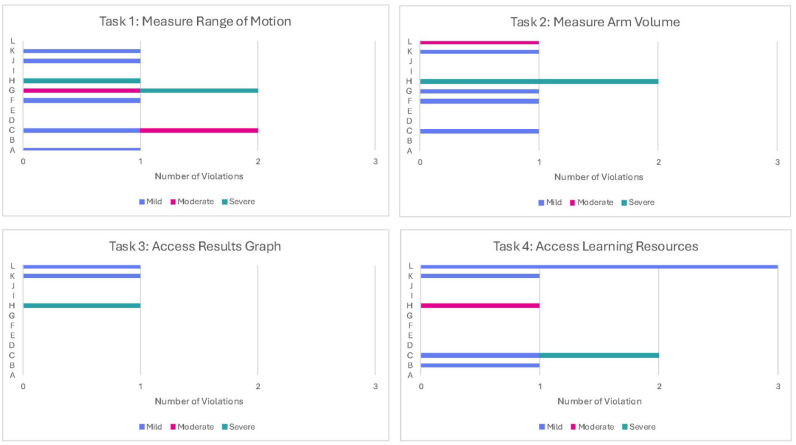
Heuristic violations by task.^a^ ^a^A = visibility of system status; B = user control and freedom; C = consistency and standards; D = error prevention; E = minimized memory loads; F = flexibility and efficiency of use; G = helping users recognize, diagnose, and recover from errors; H = help and documentation; I = leveraging interactivity; J = providing accurate, colloquial, comprehensive, and succinct content; K = providing tailored, flexible, and layered content; L = using visuals to complement text but avoiding tables.

Figure 4 provides information on the number of heuristic violations by task. The task that accumulated the greatest number of unique violations across all heuristics (n = 9) and the most moderate to severe violations (n = 4) was Task 1 (Measure ROM). Raters recommended adding an auditory signal at the start and stop of measurements to improve accuracy. Task 2 (Measure Arm Volume) received 7 violations, mostly mild (n = 4), with 2 attached to the heuristic “help and documentation.” The heuristic “using visuals to complement text but avoiding tables” received a moderately rated violation in this task because one rater felt the explanatory videos were difficult to see on a phone. Task 3 (Access Results Graph) was given the least number of violations (n = 3), while task 4 (Access Resources) had 8 unique violations. Within this task, most concerns (n = 3) related to the heuristic criteria of “use visuals to complement text, but avoid tables” with concerns about difficulty visualizing the bottom text due to the menu bar obstructing the view, an unclear exercise photo, and incomplete component of an exercise photo.

Health providers identified several strengths of the mHealth system on the provider survey. This included: easy to use (simple navigation), clear instructions (easy to understand content), inclusion of videos / graphics to explain content in each task (good length and pace of instruction), pleasing esthetics (good use of colors), inclusion of progress graph to help users see change over time, and ability to tap anywhere on screen for ROM measurement. Areas for improvement included: challenges measuring ROM (for external rotation some questioning the measurements accuracy, noting that changes in screen orientation may alter measurement reading, and tilt of phone may alter reading), system function issues (home toggle button on bottom of screen covering content, some images and font small on phone), need for additional functions (add audible beep when activating/deactivating ROM measurement, note to facilitate the remeasuring of ROM if reading greater than expected/possible), and the need for additional content (more photos in posture section, 8 or 10 month viewing option in progress graph, movement arrows in exercise images). Providers felt that App was appropriate for anyone with breast cancer (during or after treatment, pre/post surgery, with or without UE impairment) who understood English and owned a smartphone. They believed certain features of this mHealth system could also be beneficial for other individuals with shoulder injuries, even if they do not have a breast cancer diagnosis.

## Discussion

As the number of BC survivors grows, persistent functional limitations will be an enormous public health challenge. The *StrongArms-Cancer* system was designed to support survivors of BC in maintaining UE function. It addresses 2 common BC related UE impairments, lymphedema and reduced ROM, that can be managed effectively using rehabilitation strategies if identified early. The *StrongArms-Cancer* system also provides triaged management strategies for common UE impairments. The purpose of this study was to test the usability of this mHealth system from the patient and health provider perspective. Study results demonstrated usability of the *StrongArms-Cancer* mHealth system, as measured by SUS and MAUQ scores, and identified ways to improve the App prior future phases assessing reliability and validity of the system, and thereafter it’s full implementation. Over the long term, this system has the potential to be an accessible, cost-effective way to prevent and provide early detection of lymphedema and UE ROM limitations. Over time, prevention and early identification will decrease burden on (a) the individual, as they will have higher levels of overall functioning and be able to return to meaningful activities, and (b) the healthcare system, as it will help to prevent serious UE impairments requiring management.

### Patient Perspective

The most common usability issues identified when using the *StrongArms-Cancer* mHealth system were workflow issues, specifically related to using the measurement tools associated with the system (ie, measuring ROM and using the measurement wand to measure arm volume). This is not surprising as this mHealth system is complex and integrates numerous forms of technology. Research demonstrates that as the complexity of an mHealth system increases usability issues rise due to the heightened cognitive load needed to use the system and more complicated user interfaces.^61-63^ Users often struggle to navigate complex systems, resulting in a steep learning curve and increased error rates during initial use. Since this was the participants’ first time using the *StrongArms-Cancer* system, it is not surprising they had workflow issues with the complex measurement tasks. Other common usability issues for mHealth systems cited in the literature include poorly designed user interfaces, limited customization options, and integration issues, which can frustrate users and decrease engagement and satisfaction,^61,64^ however these issues were not revealed in our evaluation of the *StrongArms-Cancer* mHealth system. To improve the *StrongArms-Cancer* system we have addressed issues with workflow, as well as color, understanding instructions, graphics, layout, font, iconography/terminology, applicability, and timeliness. Modifications made included adjusting color (to make important buttons/icons brighter and differentiate content); adding more blank space in the resource section; increased font size and icon size throughout the App, adding text with navigation icons; adding an audible beep with the start/stop of ROM; adding easy glide material to the measurement wheel; slowing down the movement indicator on the measurement wand; creating a separate exit/log out button, and adding a new frequently asked questions button on the home screen.

Despite the usability issues identified in this trial, SUS and MAUQ scores were high (SUS average score 86.9; MAUQ mean items scores 6.5/7). The SUS score constitutes an “excellent” usability score and demonstrates strong perception of usability with the mHealth system.^54^ While we did not analyze SUS scores based on age, research has suggested the SUS scores of the same mHealth system decrease with age, therefore this is an area of future research for this system.^54^ The MAUQ items score average of 6.5 demonstrates high usability based on ease of use, interface/satisfaction, and usefulness.^51^ Based on these results and our pre-defined criteria to demonstrate usability (SUS scores >70^53^ and MAUQ items scores >5/7^51^), testing demonstrated strong usability of the *StrongArms-Cancer* system, and we will proceed to the next stages of system evaluation. Further, although we only conducted these outcome measures once, since this was the first time users engaged with this mHealth system, we believe that scores on both scales would increase if measured after modifications are made based on user feedback.

### Provider Perspective

The most common heuristic violation identified by clinicians related to “help and documentation,” specifically the need to include a FAQ section and provide additional help for users with additional questions. Common heuristic violations described in the literature for other mHealth systems include inadequate visibility of system status and inconsistent terminology that can confuse users. Many apps are said to also lack error prevention features and easy navigation.^65,66^ While this mHealth system was created to be a patient-facing self-management tool to use at home, there may be some benefit of integrating an “ask a therapist” feature in the future. This feature would provide users with direct access to professional medical advice, which is known to enhance patient engagement and confidence in managing their health. However, challenges would include staffing this feature, ensuring timely responses from the clinicians involved, and maintaining quality and accuracy of information without ensuring a formal assessment has taken place.^67-69^ While there is the potential for artificial intelligence (AI) models to fill this role in the future, it is also important to manage user expectations regarding the limitations of digital medical advice.^67-69^ This is an important area for future research. In the follow-up heuristic survey, clinicians stated that strengths of the App included simple navigation and easy to understand content, which is important from a health literacy perspective. Areas for improvement mentioned in the survey were consistent with participant feedback with the addition of suggestions for additional content, and suggestions to ensure accuracy of ROM measurements for users. In response to this feedback, modifications have been made to the *StrongArms-Cancer* mHealth system, including the addition of information related to precisely measuring ROM (eg, locking home screen orientation, and a prompt to remeasure if measured values are much higher than expected normal values), the modification of content in the resource section (eg, additional photos or updates to photos to clarify movements and reordering of content), and the addition of content (eg, in the “posture” and “strength training” sections). Clinicians felt the system had wide applicability to help as a self-management tool for all individuals with breast cancer across the cancer trajectory. Therefore, increasing accessibility to the system will be a primary goal of commercialization into the future.

### Future Research

It’s important to highlight that clinical validity, diagnostic accuracy, and measurement reliability were not evaluated within the scope of the present study. Prior to commercialization efforts, the next phase of evaluation of this mHealth system will be reliability and validity testing of the measurement tools (goniometer to measure ROM and measurement wand to measure arm volume) integrated within the system. This will involve having participants self-measure ROM and arm volume using the mHealth system, and also having these same measurements performed by a trained therapist over multiple time points. This will enable us to determine the accuracy and consistency of the measurement functions which is of utmost importance in a self-care tool to prevent false positive readings.

### Limitations

While there are numerous strengths to this study, there are several limitations, which are important when interpreting results. First, this study has limitations that may impact generalizability. All participants were drawn exclusively from Ontario, Canada, which limits the applicability of the results to broader populations, particularly outside this region or in different healthcare settings. Further, as the usability of mHealth systems is highly context-dependent, it’s important to note that the findings of this project primarily reflect populations with relatively high levels of health and digital literacy. Future studies should consider evaluating the system in diverse cultural or healthcare settings to better understand its broader applicability to various populations. Additionally, the use of a convenience sample of clinicians for the heuristic evaluation raises the possibility of selection bias, as those who agreed to participate may not represent the wider healthcare community. Furthermore, the exclusion of individuals who did not speak or understand English may have resulted in a loss of valuable insights from diverse cultural backgrounds and language fluency levels, further limiting the applicability of the findings. These factors highlight areas for future research to enhance representativeness and inclusivity.

## Conclusion

This project aimed to evaluate the usability of the *StrongArms-Cancer* mHealth system from both patient and healthcare provider perspectives. The findings from think-aloud sessions and heuristic evaluations revealed high user satisfaction and demonstrated effective usability. Consequently, the system shows promise in aiding the proactive surveillance and monitoring of individuals with a history of breast cancer. Early detection of impairments can facilitate timely treatment, leading to better prognoses and preventing disability, which could significantly reduce the incidence of chronic UE impairments in individuals with BC while lowering healthcare costs associated with treating these secondary complications. This self-care mHealth system may empower survivors to monitor UE function and act promptly on meaningful changes. Future research should focus on developing cancer-specific, tailored technologies that leverage AI for self-monitoring of impairments, ultimately optimizing outcomes for individuals affected by cancer.

## Supplemental Material

sj-docx-1-inq-10.1177_00469580261441759 – Supplemental material for Monitoring Upper Extremity Function of Individuals With Breast Cancer: Development and Usability of the StrongArms-Cancer mHealth System Supplemental material, sj-docx-1-inq-10.1177_00469580261441759 for Monitoring Upper Extremity Function of Individuals With Breast Cancer: Development and Usability of the StrongArms-Cancer mHealth System by Jenna Smith-Turchyn, Jordon L. Hvizd, Edward R. Sykes, Som D. Mukherjee, Tara Packham, Margaret L. McNeely, Carolyn Moorlag, Ethan Shen, Haruna Isah, Christopher Anand and Julie Richardson in INQUIRY: The Journal of Health Care Organization, Provision, and Financing

sj-docx-2-inq-10.1177_00469580261441759 – Supplemental material for Monitoring Upper Extremity Function of Individuals With Breast Cancer: Development and Usability of the StrongArms-Cancer mHealth System Supplemental material, sj-docx-2-inq-10.1177_00469580261441759 for Monitoring Upper Extremity Function of Individuals With Breast Cancer: Development and Usability of the StrongArms-Cancer mHealth System by Jenna Smith-Turchyn, Jordon L. Hvizd, Edward R. Sykes, Som D. Mukherjee, Tara Packham, Margaret L. McNeely, Carolyn Moorlag, Ethan Shen, Haruna Isah, Christopher Anand and Julie Richardson in INQUIRY: The Journal of Health Care Organization, Provision, and Financing

sj-pdf-3-inq-10.1177_00469580261441759 – Supplemental material for Monitoring Upper Extremity Function of Individuals With Breast Cancer: Development and Usability of the StrongArms-Cancer mHealth System Supplemental material, sj-pdf-3-inq-10.1177_00469580261441759 for Monitoring Upper Extremity Function of Individuals With Breast Cancer: Development and Usability of the StrongArms-Cancer mHealth System by Jenna Smith-Turchyn, Jordon L. Hvizd, Edward R. Sykes, Som D. Mukherjee, Tara Packham, Margaret L. McNeely, Carolyn Moorlag, Ethan Shen, Haruna Isah, Christopher Anand and Julie Richardson in INQUIRY: The Journal of Health Care Organization, Provision, and Financing

sj-pdf-4-inq-10.1177_00469580261441759 – Supplemental material for Monitoring Upper Extremity Function of Individuals With Breast Cancer: Development and Usability of the StrongArms-Cancer mHealth System Supplemental material, sj-pdf-4-inq-10.1177_00469580261441759 for Monitoring Upper Extremity Function of Individuals With Breast Cancer: Development and Usability of the StrongArms-Cancer mHealth System by Jenna Smith-Turchyn, Jordon L. Hvizd, Edward R. Sykes, Som D. Mukherjee, Tara Packham, Margaret L. McNeely, Carolyn Moorlag, Ethan Shen, Haruna Isah, Christopher Anand and Julie Richardson in INQUIRY: The Journal of Health Care Organization, Provision, and Financing
