# Supplementary material for: Monitoring Upper Extremity Function of Individuals With Breast Cancer: Development and Usability of the StrongArms-Cancer mHealth System
Source: Inquiry. 2026 Apr 10;63:00469580261441759. doi: 10.1177/00469580261441759 (PMC13077146; doi:10.1177/00469580261441759)
Supplement: sj-docx-2-inq-10.1177_00469580261441759 – Supplemental material for Monitoring Upper Extremity Function of Individuals With Breast Cancer: Development and Usability of the StrongArms-Cancer mHealth System [file sj-docx-2-inq-10.1177_00469580261441759.docx]

**Supplementary File 1:** Heuristic Evaluation Form

**Task:**

| **Heuristic** | **Example** | **OK**  **(✓)** | **Violation**  **(✓)** | **Severity***  **(1-3)** | **Screen Shot(s)**  **(PrtScn then paste)** | **Comment/ Recommendation** |
| --- | --- | --- | --- | --- | --- | --- |
| 1. ^†^Visibility of system status: The user is informed as to the state of the system at any given moment. | A system status indicator; the user knows if an operation was successfully completed or what additional steps are needed to complete the task |  |  |  |  |  |
| 1. ^†^User control and freedom: The user should feel in control of the system. | Clearly marked exits; users can undo or redo an action; it is difficult to perform irreversible actions. |  |  |  |  |  |
| 1. ^†^Consistency and standards: The user interface and basic system operations should be consistent. | The general layout and position of menus, buttons and other controls is consistent throughout the system. |  |  |  |  |  |
| 1. ^†^Error prevention: The interface is designed to prevent errors from occurring. | Users are presented with a confirmation option before they commit to an action. |  |  |  |  |  |
| 1. ^†^Minimize memory load: The user interface supports recognition rather than recall. | Users should not have to remember exact details. Instructions for use of the system should be visible or easily retrievable whenever appropriate. |  |  |  |  |  |
| 1. ^†^Flexibility and efficiency of use: The user interface should be customizable and flexible for different types of users. | Allow experienced users to create shortcuts for common operations; allow users to set their own preferences. |  |  |  |  |  |

|  |  |  |  |  |  |  |
| --- | --- | --- | --- | --- | --- | --- |
| **Heuristic** | **Example** | **OK**  **(✓)** | **Violation**  **(✓)** | **Severity***  **(1-3)** | **Screen Shot(s)**  **(PrtScn then paste)** | **Comment/ Recommendation** |
| 1. ^†^Help users recognize, diagnose and recover from errors: The system provides a clear and easy to understand way of recovering from an error. | Error messages are phrased in clear and meaningful language; if users make mistakes, there are obvious ways to correct them. |  |  |  |  |  |
| 1. ^†^Help and documentation: Help should be available to users when needed. | The system provides topic-specific help or FAQs. |  |  |  |  |  |
| 1. ^‡^Leverage interactivity. | Offer interactive tools (i.e. quizzes, questionnaires, glossaries, tutorials) to engage with the information and provide performance feedback. Allow users to share information with others. |  |  |  |  |  |
| 1. ^‡^Provide accurate, colloquial, comprehensive, succinct content. | Written information should be brief, relevant, and in users’ vernacular. |  |  |  |  |  |
| 1. ^‡^Provide tailored, flexible, layered content. | Prioritize information according to importance. If possible, personalise information. Provide succinct summaries but allow users to access more detailed information. Offer content in multiple languages. |  |  |  |  |  |
| 1. ^‡^Use visuals to complement text, but avoid tables. | Visuals (i.e. pictures, videos, animations) may enhance written information. If unavoidable, tables should be designed as independent, simplistic representations of information. |  |  |  |  |  |
