# Supplementary material for: Monitoring Upper Extremity Function of Individuals With Breast Cancer: Development and Usability of the StrongArms-Cancer mHealth System
Source: Inquiry. 2026 Apr 10;63:00469580261441759. doi: 10.1177/00469580261441759 (PMC13077146; doi:10.1177/00469580261441759)
Supplement: sj-pdf-3-inq-10.1177_00469580261441759 – Supplemental material for Monitoring Upper Extremity Function of Individuals With Breast Cancer: Development and Usability of the StrongArms-Cancer mHealth System [file sj-pdf-3-inq-10.1177_00469580261441759.pdf]

McMaster University HEALTH SCIENCES

School of Rehabilitation Science REACHING FURTHER

System Usability Scale

ID: ______________________________

Date: ____________________________

Part 1:

Please score the following 10-items with one of five responses that range from Strongly Disagree to Strongly Agree, in relation to your experience with the *StrongArms-Cancer* App.

Item	Strongly Disagree	Disagree	Neutral	Agree	Strongly Agree
1. I think that I would like to use this app frequently	1	2	3	4	5
2. I found the app unnecessarily complex	1	2	3	4	5
3. I thought the app was easy to use	1	2	3	4	5
4. I think that I would need regular support from a technical person to be able to use this app	1	2	3	4	5
5. I found the various functions in this app were well integrated	1	2	3	4	5
6. I thought there was too much inconsistency in this app	1	2	3	4	5
7. I would imagine that most people would learn to use this app very quickly	1	2	3	4	5
8. I found the app very cumbersome to use	1	2	3	4	5
9. I felt very confident using the app	1	2	3	4	5
10. I needed to learn a lot of things before I could get going with this app	1	2	3	4	5

Part 2:

Please score the following item with one of seven responses that range from 'Worst Imaginable' to 'Best Imaginable' in relation to your experience with the *StrongArms-Cancer* App.

11. Overall, I would rate the user-friendliness of this product as:	❑ Worst Imaginable	❑ Awful	❑ Poor	❑ OK	❑ Good	❑ Excellent	❑ Best Imagineable
