# Supplementary material for: Monitoring Upper Extremity Function of Individuals With Breast Cancer: Development and Usability of the StrongArms-Cancer mHealth System
Source: Inquiry. 2026 Apr 10;63:00469580261441759. doi: 10.1177/00469580261441759 (PMC13077146; doi:10.1177/00469580261441759)
Supplement: sj-pdf-4-inq-10.1177_00469580261441759 – Supplemental material for Monitoring Upper Extremity Function of Individuals With Breast Cancer: Development and Usability of the StrongArms-Cancer mHealth System [file sj-pdf-4-inq-10.1177_00469580261441759.pdf]

mHealth App Usability Questionnaire (MAUQ)

ID: ___________________

Date: _________________

#	Statements	N/A	1 2 3 4 5 6 7
1	The app was easy to use.	☐	DISAGREE ☐ ☐ ☐ ☐ ☐ ☐ ☐ AGREE
2	It was easy for me to learn to use the app.	☐	DISAGREE ☐ ☐ ☐ ☐ ☐ ☐ ☐ AGREE
3	The navigation was consistent when moving between screens.	☐	DISAGREE ☐ ☐ ☐ ☐ ☐ ☐ ☐ AGREE
4	The interface of the app allowed me to use all the functions (such as entering information, responding to reminders, viewing information) offered by the app.	☐	DISAGREE ☐ ☐ ☐ ☐ ☐ ☐ ☐ AGREE
5	Whenever I made a mistake using the app, I could recover easily and quickly.	☐	DISAGREE ☐ ☐ ☐ ☐ ☐ ☐ ☐ AGREE
6	I like the interface of the app.	☐	DISAGREE ☐ ☐ ☐ ☐ ☐ ☐ ☐ AGREE
7	The information in the app was well organized, so I could easily find the information I needed.	☐	DISAGREE ☐ ☐ ☐ ☐ ☐ ☐ ☐ AGREE
8	The app adequately acknowledged and provided information to let me know the progress of my action.	☐	DISAGREE ☐ ☐ ☐ ☐ ☐ ☐ ☐ AGREE
9	I feel comfortable using this app in social settings.	☐	DISAGREE ☐ ☐ ☐ ☐ ☐ ☐ ☐ AGREE
10	The amount of time involved in using this app has been fitting for me.	☐	DISAGREE ☐ ☐ ☐ ☐ ☐ ☐ ☐ AGREE
11	I would use this app again.	☐	DISAGREE ☐ ☐ ☐ ☐ ☐ ☐ ☐ AGREE
12	Overall, I am satisfied with this app.	☐	DISAGREE ☐ ☐ ☐ ☐ ☐ ☐ ☐ AGREE
13	The app would be useful for my health and well-being.	☐	DISAGREE ☐ ☐ ☐ ☐ ☐ ☐ ☐ AGREE
14	The app improved my access to healthcare services.	☐	DISAGREE ☐ ☐ ☐ ☐ ☐ ☐ ☐ AGREE
15	The app helped me manage my health effectively.	☐	DISAGREE ☐ ☐ ☐ ☐ ☐ ☐ ☐ AGREE

16	This app has all the functions and capabilities I expected it to have.	☐	DISAGREE ☐ ☐ ☐ ☐ ☐ ☐ ☐ AGREE
17	I could use the app even when the Internet connection was poor or not available.	☐	DISAGREE ☐ ☐ ☐ ☐ ☐ ☐ ☐ AGREE
18	This mHealth app provides an acceptable way to receive healthcare services, such as accessing educational materials, tracking my own activities, and performing self-assessment.	☐	DISAGREE ☐ ☐ ☐ ☐ ☐ ☐ ☐ AGREE

In this questionnaire, 1 – strongly disagree, 2 – disagree, 3 – somewhat disagree, 4 – neither agree nor disagree, 5 – somewhat agree, 6 – agree, 7 – strongly agree
